# Preclinical immunogenicity and protective efficacy of a SARS-CoV-2 RBD-based vaccine produced with the thermophilic filamentous fungal expression system *Thermothelomyces heterothallica* C1

**DOI:** 10.3389/fimmu.2023.1204834

**Published:** 2023-06-09

**Authors:** Mariana Gonzalez-Hernandez, Franziska Karola Kaiser, Imke Steffen, Malgorzata Ciurkiewicz, Geert van Amerongen, Ronen Tchelet, Mark Emalfarb, Markku Saloheimo, Marilyn G. Wiebe, Marika Vitikainen, Irina C. Albulescu, Berend-Jan Bosch, Wolfgang Baumgärtner, Bart L. Haagmans, Albert D. M. E. Osterhaus

**Affiliations:** ^1^ Research Center for Emerging Infections and Zoonoses, University of Veterinary Medicine Hannover, Foundation, Hannover, Germany; ^2^ Institute for Biochemistry, University of Veterinary Medicine Hannover, Foundation, Hannover, Germany; ^3^ Department of Pathology, University of Veterinary Medicine Hannover, Foundation, Hannover, Germany; ^4^ Viroclinics Xplore, Schaijk, Netherlands; ^5^ Dyadic International, Inc., Jupiter, FL, United States; ^6^ VTT Technical Research Centre of Finland Ltd., Espoo, Finland; ^7^ Virology Section, Infectious Diseases and Immunology Division, Department of Biomolecular Health Sciences, Faculty of Veterinary Medicine, Utrecht University, Utrecht, Netherlands; ^8^ Department of Viroscience, Erasmus Medical Center, Rotterdam, Netherlands

**Keywords:** SARS-CoV-2, receptor-binding domain, vaccine, hamster, *Thermothelomyces heterothallica*, C1, filamentous fungus

## Abstract

**Introduction:**

The emergency use of vaccines has been the most efficient way to control the coronavirus disease 19 (COVID-19) pandemic. However, the emergence of severe acute respiratory syndrome coronavirus 2 (SARS-CoV-2) variants of concern has reduced the efficacy of currently used vaccines. The receptor-binding domain (RBD) of the SARS-CoV-2 spike (S) protein is the main target for virus neutralizing (VN) antibodies.

**Methods:**

A SARS-CoV-2 RBD vaccine candidate was produced in the Thermothelomyces heterothallica (formerly, Myceliophthora thermophila) C1 protein expression system and coupled to a nanoparticle. Immunogenicity and efficacy of this vaccine candidate was tested using the Syrian golden hamster (Mesocricetus auratus) infection model.

**Results:**

One dose of 10-μg RBD vaccine based on SARS-CoV-2 Wuhan strain, coupled to a nanoparticle in combination with aluminum hydroxide as adjuvant, efficiently induced VN antibodies and reduced viral load and lung damage upon SARS-CoV-2 challenge infection. The VN antibodies neutralized SARS-CoV-2 variants of concern: D614G, Alpha, Beta, Gamma, and Delta.

**Discussion:**

Our results support the use of the Thermothelomyces heterothallica C1 protein expression system to produce recombinant vaccines against SARS-CoV-2 and other virus infections to help overcome limitations associated with the use of mammalian expression system.

## 1 Introduction

The ongoing COVID-19 pandemic, caused by severe acute respiratory syndrome coronavirus 2 (SARS-CoV-2), a novel member of the family *Coronaviridae*, has so far resulted in more than 600 million cases and more than 6 million deaths worldwide (1–4). Vaccination against SARS-CoV-2 with several new-generation vaccines has successfully reduced the spread and impact of COVID-19 (5–7). Therefore, the World Health Organization has advocated vaccination as the best way to combat the ongoing pandemic (8). Given that the spike (S) protein of coronaviruses is the main target for virus neutralizing (VN) antibodies, vaccine development efforts have largely focused on this viral protein (9, 10). The SARS-CoV-2 S protein mediates viral attachment and entry. The S protein consists of two subunits: the S1 domain harboring the receptor-binding domain (RBD) and the S2 domain harboring the fusion peptide (11). Currently, the most frequent vaccine platforms used worldwide against SARS-CoV-2 are mRNA, recombinant adenovirus, and subunit vaccines, based on the expression of the S protein. Unfortunately, limited production capacity, high cost of goods, and logistic hurdles limit their effective use worldwide. Furthermore, largely because of escape mutations arising in the S protein, global circulation of SARS-CoV-2 variants in a partially immune population continues to raise challenges in containing the ongoing pandemic (12). Alternative production platforms that do not suffer from these limitations may offer opportunities for the development of COVID-19 vaccines that can more effectively be used in low- and middle-income countries. The protein expression technology used for vaccine development in the present study is based on the use of the *Thermothelomyces heterothallica fungus* (C1). It offers a system that uses less sophisticated media and fermentation technology and produces higher yields at lower cost than mammalian cell based systems (13, 14). This technology may provide a new avenue toward a global vaccination strategy. Here, we have used the Syrian golden hamster model to evaluate the immunogenicity and efficacy of SARS-CoV-2 RBD-based vaccine candidates produced with the *Thermothelomyces heterothallica* C1 protein expression system.

## 2 Materials and methods

### 2.1 Virus

SARS-CoV-2 (614G isolate BetaCoV/Munich/BavPat1/2020; European Virus Archive Global #026 V-03883; kindly provided by Dr. C. Drosten) was obtained from a clinical case in Germany diagnosed after returning from China and propagated on Vero E6 cells as previously described (15). Following three passages, the virus stock was sequenced, and no major variants (> 10%) were detected (15). All virus handling was performed in a Class II Biosafety Cabinet under Biosafety level 3 (BSL-3) conditions.

### 2.2 Production of SARS-CoV-2 RBD in C1 fungus expression systems

A DNA sequence coding for C1 endogenous CBH1 signal sequence, residues 333 to 527 of the Spike (S1) glycoprotein from SARS-CoV-2 Spike S1, a Gly/Ser linker, a SpyTag sequence of 13 amino acids, a Gly/Ser-linker, and C-tag (EPEA) flanked by homologous recombination sequences to the C1-cell DNA expression vector and MssI restriction enzyme sites was designed (**Supplementary Figure 1**) and synthesized by GenScript (Piscataway, New Jersey, USA). The codon usage was optimized for expression in *Thermothelomyces heterothallica*, and the construct was cloned as described in Espinosa et al. (2021) (16), Lazo et al. (2022) (17), and Nechooshtan et al. (2022) (18). Production strains for the RBD-Spytag were generated in the C1 strain DNL155 with 14 deletions of native protease genes as described before (16). Fermentations were carried out at 38°C, pH 6.8, for 5 days as previously described (16).

RBD-Spytag-C-tag was purified by affinity chromatography on a CaptureSelect™ C-tag affinity matrix (Thermo Fisher Scientific) as described in Espinosa et al. (2021) and Lazo et al. (2022) (16, 17). The product obtained was 99% pure, free of fungal debris, similar as the RBD without spytag (18). The binding activity of C1-produced and C-tag affinity–purified RBD-Spytag-C-tag to human angiotensin-converting enzyme 2 (ACE2) was studied in enzyme-linked immunosorbent assay (ELISA). A microtiter ELISA plate was coated with recombinant human ACE2 receptor (SinoBiological), and a dilution series of RBD-Spytag protein was applied on wells. Bound RBD was detected by Capture Select Biotin Anti-C-tag conjugate (ThermoFisher), and the secondary detection agent was Streptavidin-HRP (Cytiva). 3,3′,5,5′-Tetramethylbenzidine (TMB) substrate was added and hydrolyzed in a colorimetric reaction. The amount of hydrolyzed substrate is proportional to the concentration of the RBD-Spytag protein present in wells. Hydrolysis reaction was stopped with sulfuric acid, A450 nm was measured, and results were analyzed by 4-parameter logistic analysis. C1-produced RBD-C-tag without a Spytag was used as a comparison.

### 2.3 Coupling of C1-produced RBD to nanoparticles

The nanoparticle mutant i301 aldolase (mi3) was used for coupling to C1-produced RBD (19). SpyCatcher-mi3-Ctag recombinant protein and mi3-SpyTag-StrepTag were expressed in Rosetta *E. coli* bacteria from ET28a (https://www.addgene.org/112255/) and pGEX-2T (GE Healthcare Life Sciences). When bacterial cultures reached an OD_600_ of ~0.8, the expression was induced with 0.5 mM Isopropyl-β-D-thiogalactopyranosid (IPTG), and incubation continued overnight, at room temperature, with shaking. Approximately 16 h later, the bacteria were pelleted by centrifugation for 45 min/5°C/4,000*g* in 50-ml tubes. Each pellet was resuspended in 10 ml of Lysis buffer [50 mM 4-(2-hydroxyethyl)-1-piperazineethanesulfonic acid (HEPES), 150 mM NaCl, 0.1% Tx-100, lysozyme (0.1 mg/ml), cOmplete™ protease inhibitors) and incubated for 30–60 min on ice. Afterward, each tube was subjected to four rounds of 30-s sonication to disrupt the bacteria. Unlysed bacteria and debris were removed by ultracentrifugation for 45 min/5°C/25,000 Revolutions per minute (RPM) using a SW32Ti rotor. Proteins were purified from the supernatants using corresponding affinity resin: CaptureSelect™ C-tag Affinity Matrix (ThermoFisher Scientific) for SpyCatcher-mi3 and Strep-Tactin^®^ Sepharose^®^ resin (IBA-Lifesciences) for mi3-SpyTag, according to manufacturer’s recommendations. Concentrations of purified proteins were determined with the NanoDrop ND-1,000 spectrophotometer.

For optimizing the coupling of nanoparticles (SpyCatcher-mi2) with the RBD-SpyTag, several molar ratios were tested by incubating overnight in Dulbecco’s Phosphate Buffered Saline (DPBS), without calcium and magnesium (Lonza), at room temperature. The filtered product was free of fungi and bacteria. The molar ratio of 1:3 RBD : Nanoparticle (NP) offered the best coupling efficiency.

### 2.4 SDS-PAGE and Coomassie staining of antigens

From each mix, 25 µl (equivalent of 2.5 µg of RBD in lane 1) was mixed with 4x Laemmli sample buffer, heat-denatured for 10 min, and separated on a continuous polyacrylamide gel (prepared in house from 4%, 10%, and 16% acrylamide solutions) in running buffer containing 25 mM Tris base, 190 mM glycine, and 0.1% Sodium dodecyl sulfate (SDS). Afterward, the gel was fixed with a solution of 50% methanol and 10% acetic acid in water for 30 min. The fixative was removed and the Coomassie Brilliant Blue G-250 staining solution (Bio-Rad) was added for an hour, following distaining in water overnight. The gel was scanned with the LI-COR Odyssey Imaging System.

### 2.5 Animal experiment

Approval for the experiment was given by the Dutch Centrale Commissie Dierproeven (CCD) (project license number 27700202114492-WP12). Ten-week-old male Syrian golden hamsters (*Mesocricetus auratus*) were divided into eight groups with five animals each, to evaluate the pre-clinical efficacy of the RBD-based vaccine. At day 0, serum samples were collected, and the hamsters were injected intramuscularly with Phosphate Buffered Saline (PBS) (control group), 10 µg of SARS-CoV-2 RBD, 10 µg of SARS-CoV-2 RBD coupled to a nanoparticle (RBD-nano), and 10 µg of SARS-CoV-2 RBD with the non-coupled nanoparticle. As adjuvant, aluminum hydroxide (alum) 2% (Croda GmbH) was used in a 2:1 antigen:alum ratio. We also evaluated the antibody response of 10 µg of SARS-CoV-2 RBD, RBD-nano, and the RBD plus the nanoparticle in combination with alum as adjuvant. Hamsters received a boost with the respective vaccine candidates 28 days after the first dose. Hamsters were challenged intranasally with 10^4^ Tissue Culture Infectious Dose (TCID_50_) of SARS-CoV-2 614G strain 42 days after the first dose of vaccination. Four days after infection, the animals were humanely euthanized, necropsy was conducted, and tissues were collected for further processing.

### 2.6 RT-qPCR

Viral RNA was extracted using a QIAmp Viral extraction kit according to the manufacturer’s instructions. Reverse transcription-real-time polymerase chain reaction (RT-qPCR) assay was performed using the protocol established by the Institut Pasteur (20). In brief, primers and probe targeting SARS-CoV-2 RdRp gene were used following the Super Script III Platinum One-Step RT-qPCR (Invitrogen) protocol. Amplification was performed as followed: reverse transcription at 55°C for 20 min, denaturation at 95°C for 3 min, followed by 50× cycles of amplification at 95°C for 15 s and at 58°C for 30 s where data were acquired. Further analysis and Quantification Cycle (Cq) values were determined using the Bio-Rad CFX Maestro software (Bio-Rad).

### 2.7 Virus infectivity titration

Infectious SARS-CoV-2 in lung and nasal turbinate tissues was quantified in Vero cells (American Type Culture Collection (ATCC) CCL-81) in 96-well plates, as previously described (5, 21). In short, 10-fold serial dilutions of homogenized tissues were used to infect the cells, starting dilution 100- and 10-fold for lung and nasal turbinate homogenate, respectively. Plates were incubated in a humidified atmosphere, at 37°C, 5% CO_2_. Cytopathic effect was evaluated 5 days after infection. Virus titers (TCID_50_/ml) were calculated using the Spearman–Karber method.

### 2.8 SARS-CoV-2 RBD ELISA

Antibodies against SARS-CoV-2 RBD were detected *via* an in-house IgG ELISA, as previously described (22). In short, 96-well microtiter ELISA plates were coated with SARS-CoV-2 Wuhan-Hu-1 RBD protein in PBS. Plates were incubated and blocked with 1% skimmed milk powder in PBS. After 1 h of incubation at 37°C, a 1:50 dilution of the serum samples was added, and plates were incubated, washed, and incubated with a 1:4,000 dilution of goat anti-Syrian hamster Immunoglobulin G (IgG) H&L conjugated to horse radish peroxidase (Abcam). Next, plates were washed, and TMB substrate (Invitrogen) was added. Finally, after incubation, 2 M H_2_SO_4_ was added to stop the reaction, and an optical density at 450 nm was measured using a Tecan Infinite 200 Microplate reader (Tecan).

### 2.9 Virus neutralization assay

VN antibodies present in the sera were detected as described previously (22, 23). In short, inactivated serum samples (at 56°C for 30 min) were first diluted 1:10 followed by two-fold serial dilutions. Vero cells (ATCC CCL-81) were seeded in a 96-well tissue culture plate. Diluted sera were mixed with 200 TCID_50_ of SARS-CoV-2 Wuhan-Hu-1 and incubated for 1 h at 37°C. Serum–virus mix was added to a monolayer of Vero cells and further incubated at 37°C, 5% CO_2_. After 8 h of incubation, cells were fixed using 4% Paraformaldehyde (PFA) and incubated for 30 min at room temperature. Next, PFA was removed, and cells were incubated for 15 min with 80% methanol. For staining, plates were blocked using 1% BSA in PBS–0.05% Tween 20 for 30 min at 37°C. SARS-CoV-2 was detected using a 1:1,000 dilution of rabbit polyclonal anti–SARS-CoV-2 nucleocapsid (Sino Biological). After 1 h of incubation at 37°C, cells were washed with PBS–0.05% Tween 20 and incubated with a 1:1m000 dilution of anti–rabbit–IgG–Alexa Flour 488 (Invitrogen). Finally, cells were washed twice with PBS–0.05% Tween 20. Fluorescent cells were counted using the C.T.L. S6 Ultimate-V Analyzer, and data were analyzed using CTL ImmunoSpot^®^ software. Neutralizing antibody titers are expressed as the dilution that gave a 50% reduction of stained cells (NT50).

### 2.10 Rhabdoviral pseudotype particles for virus neutralization assay

Pseudotyped vesicular stomatitis virus (VSV) particles bearing the S protein of SARS-CoV-2 variants of concern (VOCs) were prepared as described previously (5). In short, replication-deficient VSV that encodes for enhanced fluorescent protein and firefly luciferase (VSV*ΔG-GFP-FLuc) and plasmids that encode for SARS-CoV-2-S Wuhan, D614G, Alpha, Beta, Gamma, Delta, Omicron BA.1, and Omicron BA.5 variants were provided by Stefan Pöhlmann. 293T cells were transfected with the desired S protein or empty vector as control. Cells were infected with VSV*ΔG-GFP-Fluc 24 h after transfection and incubated 1 h at 37°C. Then, cells were washed three times with PBS, a 1:1,000 dilution of supernatant from I1-hybridoma cells (ATCC, CRL-2700) was added, and cells were incubated for 1 h at 37°C. Next, cells were washed once with PBS, and fresh medium was added. Supernatant was collected after an incubation period of 16–18 h, cellular debris was removed by centrifugation (4,500 RPM, 10 min), and aliquots of clarified supernatant were prepared and stored at −80°C until use. Pseudotyped concentration was calculated by TCID_50_. VN antibodies against SARS-CoV-2 VOCs were performed as follows: Serum samples were initially diluted 1:10, followed by 1:2 serial dilutions until a 1:2,560 dilution. Each serum sample was mixed with 200 TCID_50_ of each variant and incubated for 1 h at 37°C. Afterward, serum-pseudotype particle mix was added to Vero cells. Luciferase activity was measured as indication for transduction efficiency after 16–18 h of incubation. For this, cells were lysed using Lysis-Juice (PJK) according to the manufacturer’s instructions. Next, cell lysates were transferred to a white 96-well plate, and firefly luciferase activity was measured by using Beetle-Juice substrate (PJK) and a Tecan Infinite 200 Microplate reader.

### 2.11 Immunohistochemistry analysis

For histopathology, left lung lobes were fixed by injection and immersion with 10% buffered formalin. Tissues were subsequently embedded in paraffin and cut into 2-µm-thick sections. Lesions were evaluated on hematoxylin and eosin (HE)–stained sections with a semiquantitative scoring system described previously (24), with mild modifications. Alveolar inflammation was scored as follows: 0 = no lesion; 1 = minimal, occasional small foci, less than 1% of tissue affected; 2 = mild, 2%–25%; 3 = moderate, 26%–50%; 4 = severe, 51%–75%, 5 = subtotal, >75% of tissue affected. In addition, the presence or absence of alveolar edema, hemorrhage, necrosis/fibrin exudation, and pneumocyte type II hyperplasia was recorded (0 = not present; 1 = present). Inflammatory infiltrates and necrosis in the airways (bronchi and bronchioli) were scored as follows: 0 = no lesion; 1 = minimal, occasional small foci of inflammation, less than 1% of tissue affected; 2 = mild, 2%–25%; 3 = moderate, 26%–50%; 4 = severe, 51%–75%; 5 = subtotal, >75% of tissue affected. In addition, the presence or absence of epithelial necrosis, hyperplasia and intraluminal exudate was recorded (0 = not present; 1 = present). Vascular lesions included scoring of perivascular infiltrates (0 = no; 1 = 1–2 cell layers; 2 = 3–5 cell layers; 3 = 6–10 cell layers; 4 ≥ 10 cell layers), presence or absence of vasculopathy (characterized by endothelial cell hyperplasia, endothelialitis, and mural inflammatory infiltrates), and perivascular haemorrhage (0 = not present; 1 = present) were assessed. The total score reflects the sum of all scores for the separate compartments.

Immunohistochemistry for SARS-CoV-2 nucleoprotein was performed using a monoclonal mouse primary antibody (Sino Biological, Peking, China-40143-MM05; dilution 1:16,000, incubation over night at 4°C), the Dako EnVision+ polymer system (Dako Agilent Pathology Solutions), and 3,3′-diaminobenzidine tetrahydrochloride (Sigma-Aldrich) as described previously (25, 26). The amount of viral antigen was quantified separately in the alveoli and the airways with a five-tiered semiquantitative scoring system: 0 = no antigen; 1 = minimal, occasional positive cells, less than 1% of tissue affected; 2 = mild, 2%–25%; 3 = moderate, 26%–50%; 4 = severe, 51%–75%; 5 ≥ 75% of cells immunolabeled). In addition, the presence or absence of immunolabeled cells in the bronchial/bronchiolar exudate was recorded (0 = absent; 1 = present). The combined score is the sum of the alveolar and the airways scores. The evaluation of histology and immunohistochemistry was performed by a board-certified pathologist (MC), who was blinded to the group assignment. Scoring was confirmed by a second board certified pathologist (WB).

### 2.12 Statistical analysis

The statistical significance for the different assays was analyzed using GraphPad Prism version 9 (https://www.graphpad.com).

## 3 Results

### 3.1 SARS-CoV-2 RBD production in the *T. heterothallica* C1 protein expression system and coupling to nanoparticles

**Figure 1:**
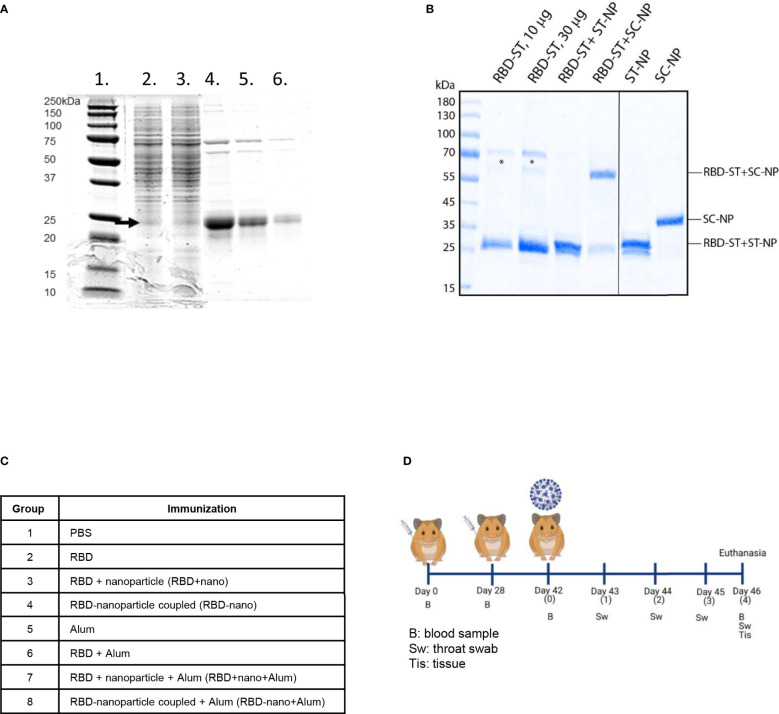
Production and analysis of antigens used for immunization. **(A)** RBD-Spytag in C1 fermentation process and C-tag affinity–purified RBD-Spytag analyzed in SDS-PAGE with Coomassie Blue dye staining. Lane 1, MW protein marker; lane 2, 98 h of fermentation supernatant; lane 3, 116 h of fermentation supernatant, which is the starting material for the affinity purification; lanes 4–6, final purified and dialyzed RBD-Spytag protein of 6.5, 3.2, and 1.3 µg, respectively, loaded on SDS-PAGE. The RBD-Spytag, corresponding to the correct size of 24-kDa protein, is marked with an arrow. **(B)** SDS-PAGE analysis was performed on the SARS-CoV-2 spike RBD antigens or RBD/nanoparticle mixtures used for immunization (left panel). For comparison, nanoparticles were also analyzed separately (right panel). Asterisks indicate contaminating yeast proteins. The abbreviations ST, SC, and NP stand for SpyTag, SpyCatcher, and nanoparticles, respectively. **(C)** Antigen combinations tested in the Syrian hamster model. **(D)** Schematic representation of the study design (Created with BioRender.com).

The RBD vaccine candidate used was based on the sequence of the RBD of SARS-CoV-2 Wuhan-Hu-1. A synthetic gene encoding the RBD fused C-terminally with Spytag was synthesized and cloned into a C1 expression vector under the *bgl8* promoter. Production strains were generated on the basis of this vector in a low-protease background C1 strain. Cultivation of the production strain in a fed-batch process for 5 days resulted in production of RBD-Spytag at the level of approximately 0.45 g/L. Single-step purification of the RBD-Spytag molecule with C-tag affinity chromatography yielded a preparate of moderate purity (**Figure 1A**) that was used in subsequent studies. ELISA for binding to the ACE2 receptor showed clear but somewhat lower binding activity as compared with a RBD preparate that was used as a control. At least part of the lower binding could be explained by the lower purity of the RBD-Spytag preparate. The RBD was conjugated to a nanoparticle, and coupling efficiency was verified using SDS-PAGE (**Figure 1B**).

### 3.2 SARS-CoV-2 RBD-nano–based vaccines induce anti-RBD and VN antibodies

**Figure 2:**
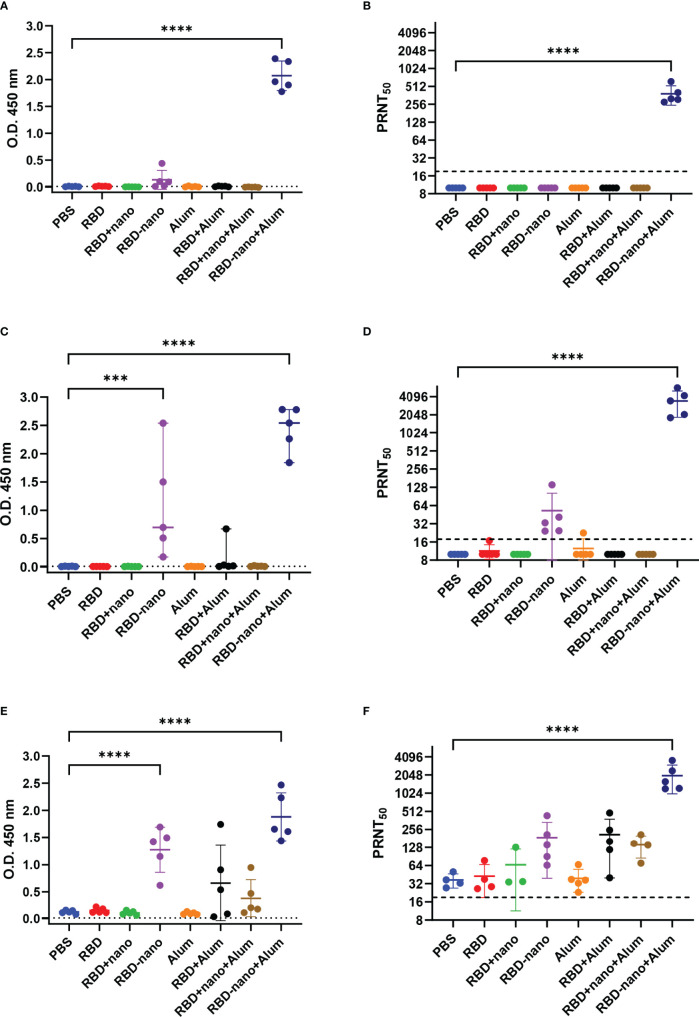
SARS-CoV-2 RBD-nano vaccine induces high neutralizing antibodies titers. Antibodies induced by the different vaccine formulations and neutralizing antibodies titers were quantified at **(A, B)** 28 days, **(C, D)** 42 days after receiving the first immunization dose, and **(E, F)** at the day of necropsy 46 days after immunization. IgG antibodies **(A, C, E)** were detected by RBD-ELISA, and dotted lines indicate the assay cutoff value $\left(\bar{x}+2SD\right)$ based on the control group. Neutralizing antibodies titers **(B, D, F)** are expressed as the reciprocal of the dilution that gave a 50% reduction of stained cells. P-values were calculated by a two-way ANOVA test; mean ± SD are presented. ****p< 0.0001; ***p< 0.001; **p< 0.01; *p< 0.05.

**Figure 3:**
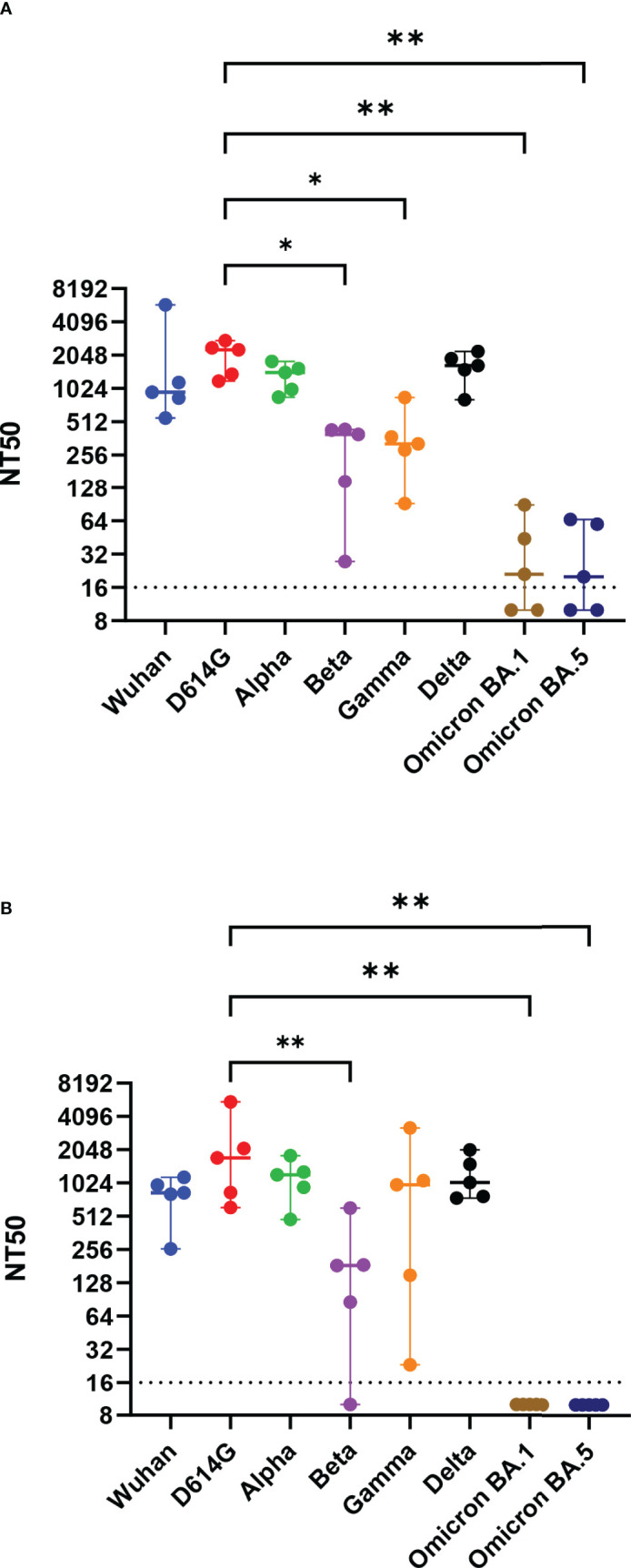
Neutralizing antibodies induced by SARS-CoV-2 RBD-nano vaccine neutralize SARS-CoV-2 VOCs. Virus neutralization assay against D614G, Alpha, Beta, Gamma, Delta, Omicron BA.1, and Omicron BA.5 variants of concern at **(A)** day of 42 and **(B)** day 46 were done using vesicular stomatitis virus pseudotyped with the respective spike protein. Titers are expressed as the reciprocal dilution that reduced entry to 50%. P-values were calculated using a one-way ANOVA analysis. ****p< 0.0001; ***p< 0.001; **p< 0.01; *p< 0.05.

Hamsters were immunized twice with RBD alone (RBD), RBD mixed with a nanoparticle (RBD + nano), or RBD coupled to a nanoparticle (RBD-nano), either with or without alum (**Figure 1C**). Serum samples were collected on day 0, day 28 (before receiving the booster dose of the vaccine), day 42 (day of challenge), and day 46 (day of necropsy) to evaluate the antibody response induced by the vaccine (**Figure 1D**). At day 28, after the first immunization, all hamsters that had received the RBD-nano with adjuvant (RBD-nano + alum) had developed detectable SARS-CoV-2 RBD serum antibodies when tested by SARS-CoV-2 RBD-based ELISA (**Figure 2A**) or virus neutralization assay (**Figure 2B**). These responses increased after receiving the second dose (**Figures 2C, D**). In contrast, none of the other groups had developed significant neutralizing antibody levels before virus challenge. After SARS-CoV-2 challenge (day 46), hamsters in the respective vaccinated groups showed variable antibody responses as revealed by RBD-based ELISA and virus neutralization assay, with still significantly higher levels (p< 0.0001) in the RBD-nano with adjuvant group compared with the control group (**Figures 2E, F**). Next, using VSV-pseudotyped with the S protein of SARS-CoV-2 VOCs, we evaluated the cross-neutralization of antibodies induced by two doses of RBD-nano + alum. Antibodies induced by RBD-nano + alum neutralized D614G, Alpha, and Delta variants to a similar extent as the Wuhan variant and, to a lesser extent, Beta and Gamma variants (**Figure 3A**). However, there was no cross-neutralization against Omicron BA.1 and BA.5. This was further confirmed when measuring neutralizing antibody responses from serum taken on day 46 (**Figure 3B**).

### 3.3 Anti–SARS-CoV-2 vaccination reduces viral titers in the lungs upon virus challenge

**Figure 4:**
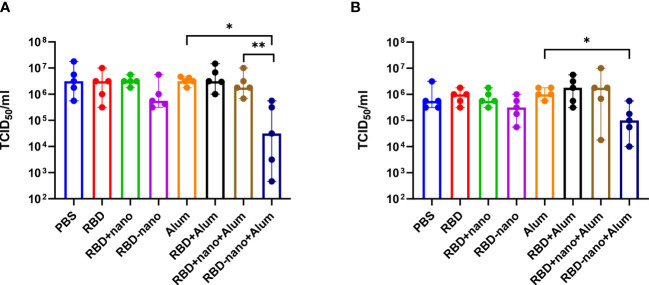
Viral load in the lung is reduced after vaccination with RBD-nano plus adjuvant. Viral titers were calculated in **(A)** lung and **(B)** nasal turbinate using TCID_50_. P-values were calculated using a Brown–Forsythe and Welch ANOVA test. ****p< 0.0001; ***p< 0.001; **p< 0.01; *p< 0.05.

To evaluate the efficacy of the vaccine candidates against virus infection, hamsters were challenged intranasally with SARS-CoV-2 (D614G), and necropsy was conducted 4 days after infection, where lungs and nasal turbinates were collected. To determine the presence of viral RNA and infectious virus in the samples, we used RT-qPCR and virus titration, respectively. A 10- to 100-fold reduction in infectivity was detected in the lungs of the groups that had received the RBD-nano without and with adjuvant, respectively (**Figure 4A**). Such differences were not found in nasal turbinate samples when compared with the control group (**Figure 4B**). However, the reduction of viral titers in lung and nasal turbinates in the group who received the RBD-nano + Alum was statistically significant (*p< 0.05) when compared with viral titers present in the group that only received Alum. At the RNA level, no apparent differences among the groups were observed (**Supplementary Figure 2**).

**Figure 5:**
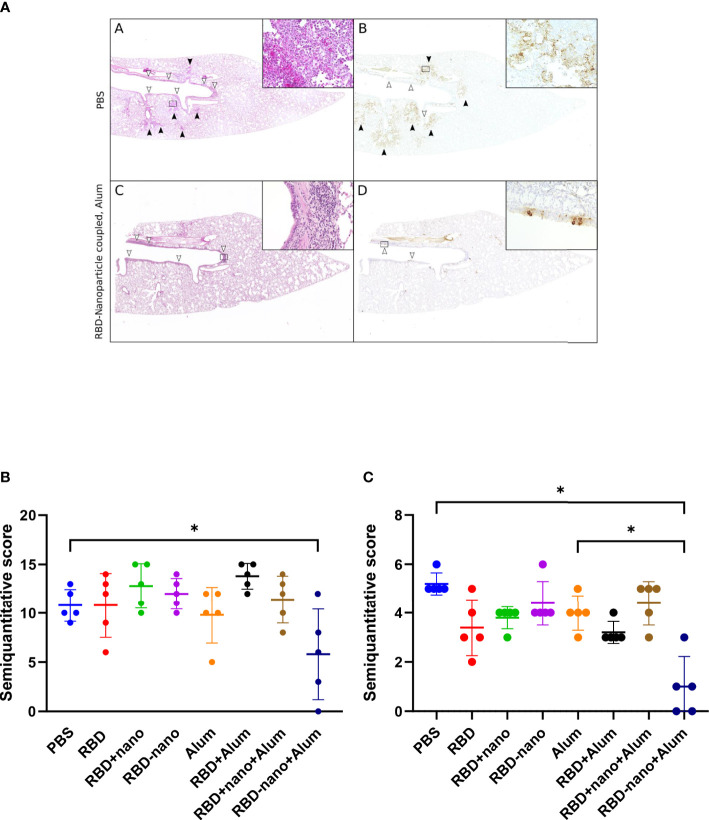
Histopathological lesions and viral antigen in the lungs of SARS-CoV-2–infected hamsters. **(A, B)** Representative images of a PBS-treated infected control hamster showing multifocal areas of inflammation in alveoli [black arrowheads and inset in **(A)**] associated with abundant viral antigen in pneumocytes [brown signal, black arrowheads, and inset in **(B)**]. Inflammatory infiltrates are also present in the airways [white arrowheads in **(A)**], but only occasional bronchial epithelial cells are positive for viral antigen [white arrowheads in **(B)**]. **(C, D)** Representative image of a hamster vaccinated with an RBD vaccine (RBD-nano + Alum) showing inflammatory lesions and viral antigen exclusively in the main airways (white arrowheads, inserts). **(A, C)** Hematoxylin and eosin stain. **(B, D)** Immunohistochemistry for SARS-CoV-2 nucleocapsid protein. Insets show 400× magnification of areas delineated by rectangles in the overview images. **(E)** Semiquantitative score of histological lesions induced by SARS-CoV-2 infection. **(F)** Semiquantitative score of SARS-CoV-2 antigen present in alveoli and airways. P-values for were calculated using a one-way ANOVA analysis. ****p< 0.0001; ***p< 0.001; **p< 0.01; *p< 0.05.

Next, lungs and nasal turbinates preserved in 10% formalin were examined for histopathological changes and viral antigen expression. We observed that hamsters immunized with the adjuvanted RBD-nano + alum showed a significant reduction in viral antigen detected in the lungs, associated with reduction of lesions (**Figure 5**), which was not the case for the nasal turbinates.

## 4 Discussion

Like other coronaviruses, the S protein of SARS-CoV-2 is highly immunogenic and the main target of neutralizing antibodies. Hence, it has been used as the main antigen for most of the vaccines developed. Depending on the platform used for vaccine production, full-length SARS-CoV-2-S–based vaccines may confer between 65% and 95% protection in humans (27). Vaccines based on mRNA expressing the S protein are among the best to induce VN antibodies and provide protection against SARS-CoV-2–associated disease. These vaccines, to varying degrees, also induce antibodies that cross neutralize newly arisen VOCs and reduce severe disease manifestations caused by these variants (28). However, some of the major disadvantages of these vaccines are the relative manufacturing complexity, high production costs, and stringent cold-chain requirements, which collectively limit their applicability, especially in low- and middle-income countries. New-generation vaccines based on purified S protein produced in a fungal expression system like the *Thermothelomyces heterothallica* C1 system would not have these disadvantages.

Here, we evaluated the immunogenicity and efficacy of a SARS-CoV-2 RBD-based vaccine candidate expressed in the C1 fungal system using Syrian golden hamsters as animal model. Our C1-RBD-Spytag–based vaccine candidate was produced at concentrations of about 0.45 g/L under so far non-optimized fungal fermentation conditions, which resulted in a product that would not require stringent cold-chain conditions. Recently, the production level of C1-RBD reached > 2 g/L in a 5-day fermentation (data not shown). Moreover, in the study done by Ramot et al., it was shown that, in New Zealand rabbits, the SARS-CoV-2 RBD produced with the C1 system, did not induce any adverse effects or systemic toxicity and induced the production IgG antibodies against SARS-CoV-2 (29). Furthermore, Lazo and colleagues proved that immunization with SARS-CoV-2 RBD produced in the C1 system induced a similar humoral response in mice as recombinant SARS-CoV-2 RBD produced in Human embryonic kidney 293 (HEK293) cells (17). We also showed that one single immunization with the RBD-nano with alum quite efficiently induced SARS-CoV-2 neutralizing antibodies to higher titers than the non-adjuvanted equivalent. Dalvie and colleagues also showed that, when using alum as an adjuvant with RBD-based viral like particles as a vaccine, the induction of VN antibodies proved to be more efficient than the CpG-adjuvanted alternative (30, 31). These results are in partial disagreement with those by Merkuleva and colleagues who evaluated the immune response induced by a trimeric uncoupled RBD-based candidate vaccine expressed in mammalian cells in different animal models. They showed a dose-dependent virus VN antibody response in the hamster model, which, however, proved to be inferior to responses found in other animal models (10). In our hands, coupling of the SARS-CoV-2 RBD to a nanoparticle proved to be crucial for the induction of high titer VN antibodies and protection, as we have shown previously for a MERS-CoV candidate vaccine in different animal species (13). It is, however, puzzling to note that, despite inducing high levels of VN antibody in the group that received the RBD-nano + alum vaccine, the reduction of virus infectivity (10- to 100-fold) in the lungs was less pronounced than the one observed when golden Syrian hamsters are pre-treated with human monoclonal antibodies against SARS-CoV-2 (5).

The reduction of virus infectivity titres in the lungs of hamsters vaccinated with candidate alum-adjuvanted RBD-nano vaccine did reach statistical significance when compared with the alum control group. This was also observed when comparing viral antigen levels (**Figure 5B**) and lesions in the lungs (**Figure 5A**). The reduction of virus infectivity titers and lesions was not observed in the nasal turbinates. Similar results have been obtained by other groups using the Syrian hamster model; Dalvie and colleagues showed that hamsters that received the RBD vaccine recovered faster than the control group (31). Moreover, in the study conducted by Chiba et al., it was shown that a SARS-CoV-2-S–based vaccine coupled to a nanoparticle conferred full protection against SARS-CoV-2 challenge in hamsters (31, 32).

Because of the continuous emergence of VOCs, it is important to know to what extent a vaccine provides cross-protection against arising virus mutants and, especially, VOCs. Therefore, we also evaluated the cross-neutralizing capacities of the hamster serum antibodies induced by the RBD-nano + alum, our most efficient vaccine candidate, against different VOCs in a VSV-based pseudotype virus neutralization assay. We showed that the induced antibodies were able to cross-neutralize early VOCs (Alpha, Beta, Gamma, and Delta). However, neutralizing antibodies present in the hamster serum were not able to cross-neutralize Omicron VOC. This indicates that, to confer better protection, the RBD needs to be exchange to the RBD of circulating VOCs. In a recent study, Walls and colleagues showed that the VN antibodies induced by an RBD vaccine or the full S protein exhibited different neutralization efficacy depending on the animal model used. Neutralizing antibodies induced in mice showed better cross neutralization against Beta and Gamma VOCs (only two-fold reduction efficiency) than VN antibodies induced in non-human primates (NHPs) that were ~6- to 8-fold less efficient in neutralizing these VOCs (33). In our study, we also observed that neutralizing antibodies induced in hamsters are affected by VOCs in a similar way as in NHP (~10-fold reduction in neutralization). Most importantly, these reduced responses against some variants, seen in NHPs and in hamsters, probably better reflect what has been observed in most human vaccine studies (28, 34, 35). Collectively, our data show that it is important to consider the way the antigen is presented, as well as the animal model in which vaccines are being tested, because the immune response observed even within hamsters appears to vary considerably from one study to another.

Together, we have shown that an adjuvanted C1 RBD-nano + alum–based vaccine induces VN antibodies, and results in reduced viral load and lung damage upon subsequent SARS-CoV-2 infection in hamsters. Given the advantages of this approach, it should be further evaluated as an alternative vaccine development strategy that may overcome some of the limitations of the current COVID-19 vaccines and vaccine candidates and, therefore, make it more applicable for low- and middle-income countries.

## Data availability statement

The raw data supporting the conclusions of this article will be made available by the authors, without undue reservation.

## Ethics statement

The animal study was reviewed and approved by Dutch Centrale Commissie Dierproeven.

## Author contributions

Production, expression, and coupling of RBD: RT, ME, MS, MW, MV, IA, and B-JB; animal experiments: MG-H, FK, and GA; pathological investigation: WB and MC; supervision: AO and BH; VNT and VSVpp: MG-H, FK, and IS; study conception and coordination: AO, BH, and RT; manuscript writing: MG-H, FK, and AO with input from all other authors. All authors contributed to the article and approved the submitted version.
