# Supplementary material for: Preclinical immunogenicity and protective efficacy of a SARS-CoV-2 RBD-based vaccine produced with the thermophilic filamentous fungal expression system Thermothelomyces heterothallica C1
Source: Front Immunol. 2023 Jun 9;14:1204834. doi: 10.3389/fimmu.2023.1204834 (PMC10289020; doi:10.3389/fimmu.2023.1204834)
Supplement: Supplementary file 1 [file DataSheet_1.docx]

Supplementary Material

Preclinical immunogenicity and protective efficacy of a SARS-CoV-2 RBD based vaccine produced with the thermophilic filamentous fungal expression system *Thermothelomyces heterothallica*, C1.

**Mariana Gonzalez-Hernandez^1†*^; Franziska Karola Kaiser^1†^, Imke Steffen^1^, Malgorzata Ciurkiewicz^2^, Geert van Amerongen^3^, Ronen Tchelet^4^, Mark Emalfarb^4^, Markku Saloheimo^5^, Marilyn G. Wiebe^5^, Marika Vitikainen^5^, Irina C. Albulescu^6^, Berend-Jan Bosch^6^, Wolfgang Baumgärtner^2^, Bart L. Haagmans^7^, Albert D.M.E. Osterhaus^1*^**

*** Correspondence:**Albert D.M.E. Osterhaus
Email: albert.osterhaus@tiho-hannover.de

Mariana Gonzalez-Hernandez

Email: mariana.gonzalez.hernandez@tiho-hannover.de

# Supplementary Figures


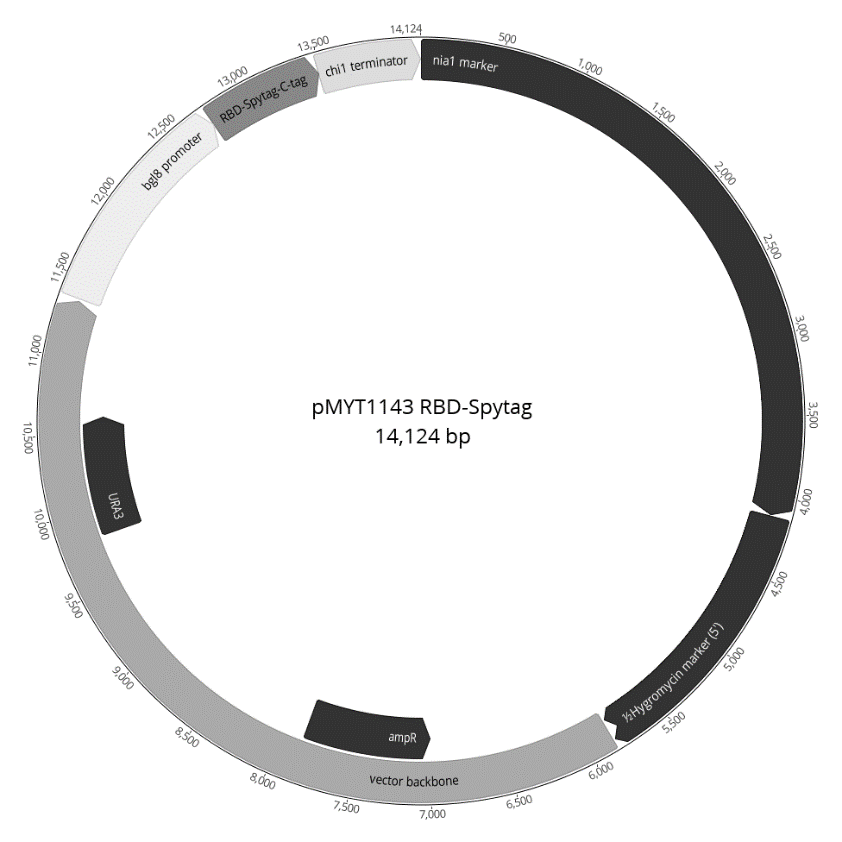


**Supplementary Figure 1.** Plasmid map of the RBD-Spytag pMYT1143. The plasmid encodes for the SARS-CoV-2 Spike S1, a SpyTag and C-tag. Two Gly/Ser linkers were added: one between the RBD/Spytag and one between the Spytag/C-tag.


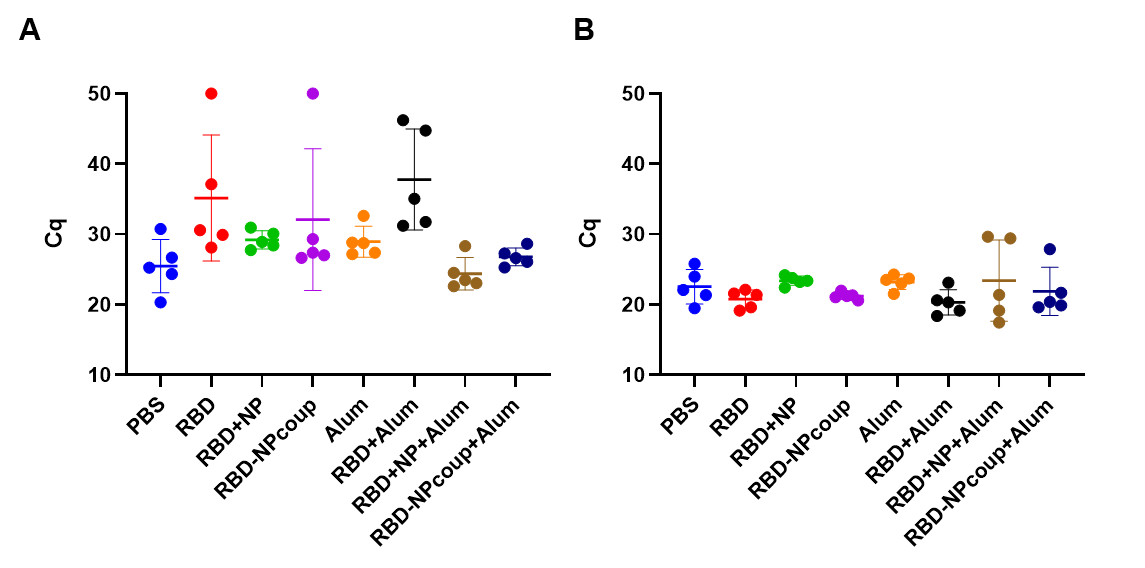


**Supplementary Figure 2.** Genomic SARS-CoV-2 present in tissue. Nasal turbinates and lung were collected at day 46 (4 d.p.i.), RNA was extracted from homogenized tissue and SARS-CoV-2 RNA was detected targeting the RdRp gene in **(A)** lung and **(B)** nasal turbinate. No significant differences were detected between groups.
